# Breath, Pulse, and Speech: A Multi‐Parameter Wearable System using Airflow‐Thermoelectric Fusion Technology

**DOI:** 10.1002/advs.202514277

**Published:** 2025-10-27

**Authors:** Zheng Zhu, Xinxin Yan, Yue Hou, Yuxuan Zeng, Zhaoyu Li, Xiaolong Sun, Chang Li, Xiaosa Liang, Qianfeng Ding, Cheng Lei, Ziyu Wang

**Affiliations:** ^1^ The Institute of Technological Sciences Wuhan University Wuhan 430072 China; ^2^ Department of Orthopedics, Renmin Hospital Wuhan University Wuhan 430072 China; ^3^ School of Power and Mechanical Engineering Wuhan University Wuhan 430072 China

**Keywords:** airflow‐thermoelectric response theory, flexible thermoelectric technology, multi‐physiological parameter fusion, wearable monitoring system, XGBoost algorithm

## Abstract

In medical emergency scenarios, conventional single‐parameter monitoring cannot fully assess patient conditions, and current technologies lack intelligent emergency‐state recognition, which delays timely treatment. To overcome these challenges, this study develops a high‐performance flexible thermoelectric textile‐based wearable system that generates a 139.7 mV open‐circuit voltage at ΔT = 30 K. To prevent skin‐thermoelectric contact from distorting sensor readings, a Kirigami spacer is integrated to ensure proper air gaps while maintaining flexibility and breathability. By integrating optical cardiac sensing, the system establishes a thermoelectric‐optical system for wireless real‐time cooperative monitoring of both respiration and cardiac activity. The research systematically investigates the effects of airflow velocity on thermoelectric output, leading to the development of an innovative dynamic airflow‐thermoelectric response theory. This theory enables speech recognition through airflow variations with 98% accuracy. Additionally, the study creates a multi‐source fusion recognition model that combines respiratory patterns, speech‐airflow, and cardiac signals to identify emergency states with 98% accuracy. These advances supplement the theoretical understanding of thermoelectric responses to physiological activities while providing decision support for emergency conditions, demonstrating considerable potential for clinical application.

## Graphical abstract

For insufficient medical emergency monitoring, this study develops a wearable system with flexible thermoelectric textiles and thermoelectric‐optical sensing. It establishes a dynamic airflow‐thermoelectric response theory (enabling 98% speech recognition) and a multi‐source fusion model for 98% emergency state recognition, aiding clinical decision‐making.

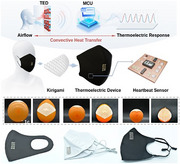

## 1 Introduction

In medical emergency response, monitoring multi‐physiological parameters is critical for patient assessment. Respiration and heartbeat, as fundamental life‐sustaining processes, serve as key clinical indicators.^[^ ^1^ ^]^ For example, they show strong distinguishing capabilities in evaluating cardiopulmonary function,^[^ ^2^ ^]^ while in metabolic disorders like diabetes, they may display irregular rhythms or abnormal patterns.^[^ ^3^ ^]^ Traditional methods often measure only one parameter at a time, limiting a comprehensive understanding of a patient's overall health.^[^ ^4^ ^]^ This limitation becomes especially problematic in emergencies such as cardiac events or falls, where patients’ physiological responses typically involve simultaneous changes across multiple parameters. These include both active distress signals like verbal communication, and passive symptoms, such as weak breathing or irregular heart rhythms.^[^ ^5^, ^6^, ^7^, ^8^ ^]^ Single‐parameter systems risk misjudgment due to incomplete data, whereas integrated monitoring of respiration, heartbeat, and speech can significantly improve early warning accuracy and provide critical time for clinical intervention. The interdependent nature of these parameters highlights the urgent need for advanced multi‐source fusion monitoring systems.

Thermoelectric devices (TEDs) have emerged as an ideal choice for physiological signal monitoring owing to their unique Seebeck effect.^[^ ^9^, ^10^, ^11^ ^]^ These devices can directly transform the temperature difference between the human body and the environment into measurable electrical signals, offering a novel technological approach for the development of wearable medical devices.^[^ ^12^, ^13^ ^]^ For example, body heat can power cardiac devices to measure ECG signals,^[^ ^14^ ^]^ while skin‐device contact changes during hand movements generate voltage variations for gesture recognition.^[^ ^15^, ^16^ ^]^ However, a long‐standing challenge lies in establishing a precise theoretical link between physiological activities and thermoelectric responses. Human activities like breathing or exercise alter device temperature differences through complex convective heat transfer, yet traditional TED research focuses primarily on material optimization and heat dissipation rather than explaining signal generation in dynamic physiological environments.^[^ ^17^, ^18^, ^19^ ^]^ Specifically, airflow‐induced heat transfer during respiratory monitoring significantly impacts TED performance,^[^ ^20^, ^21^, ^22^, ^23^ ^]^ but these mechanisms remain poorly understood. The lack of a comprehensive theoretical framework has critically hindered TED applications in physiological monitoring.

To address these challenges, this study enables the first theoretical analysis of airflow‐heat‐voltage relationships, validated through both simulations and experiments, and drives the creation of an airflow‐speech recognition system. The device employs TE cuboids (n‐type: Bi_2_Te_2.7_Se_0.3_, p‐type: Bi_0.5_Sb_1.5_Te_3_) with superior performance, achieving significantly higher output power (microwatt to milliwatt levels) compared to thin‐film (nanowatt) and textile‐based (picowatt) thermoelectric materials.^[^ ^24^, ^25^, ^26^, ^27^, ^28^, ^29^, ^30^, ^31^, ^32^ ^]^ By embedding the cube into a mask with textile electrodes, a flexible TED is developed. The Kirigami‐based isolation layer effectively prevents skin contact interference while preserving breathability. Additionally, an optical heartbeat sensor is integrated to achieve synchronized respiratory‐heartbeat monitoring, complemented by a validated cough‐alert function. The wearable system demonstrates high flexibility, waterproofing, and wireless portability, delivering 139.7 mV output voltage, 2323.21 µW power, and 3704.1 µW/cm^2^ power density at ΔT = 30 K. Further integration with deep learning enables multi‐source fusion recognition of distress speeches, abnormal breathing, and irregular heartbeats, achieving 98% accuracy in fall detection scenarios. This breakthrough validates the system's clinical utility and pioneers a novel approach for critical patient monitoring in healthcare.

## 2 Results and Discussion

### 2.1 Design of the System

**Figure 1:**
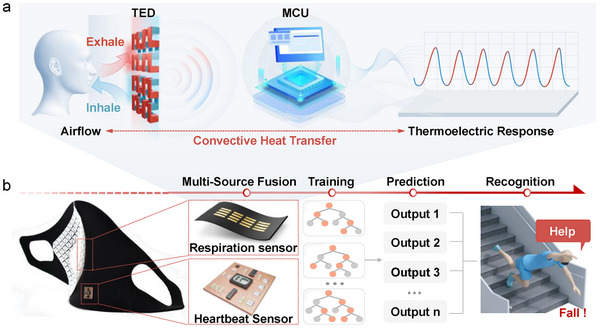
Design and mechanisms of the system. a) Respiration‐heat‐TE output voltage theory diagram and wearable respiratory‐heartbeat collaborative monitoring system. b) Multi‐source fusion for recognizing fall state.

Breathing, a crucial gas‐exchange process between the body and the external environment, involves complex physiological and physical mechanisms. As shown in **Figure** **1**a, during exhalation, intrapulmonary pressure exceeds external atmospheric pressure, which forces lung gas out through the respiratory tract, generating exhalation airflow. The exhalation airflow, at near‐body temperature, carries heat that transfers to TEDs upon contact. During inhalation, lung expansion reduces intrapulmonary pressure below the external level. Thus, inhalation air enters the lungs via the respiratory tract, forming inhalation airflow. This lower‐temperature airflow extracts heat from TEDs. Breathing's cyclic nature causes alternating convective heat transfer (CHT) between airflow and the TED, continuously changing heat distribution and temperature difference across it. Using the Seebeck effect, this temperature difference converts into a fluctuating voltage signal, which can infer respiratory intensity and frequency.

To study this theory, a wearable device was developed (Figure 1b), enabling synergistic respiratory‐heartbeat monitoring. The TED consists of rigid TE cubes (P‐type Bi_0.5_Sb_1.5_Te_3_ and N‐type Bi_2_Te_2.7_Se_0.3._ The scanning electron microscope (SEM) of the fabric electrodes and the TE cubes is depicted in Figure S1, Supporting Information), a fabric substrate, and textile electrodes, and a kirigami structure support layer, making it portable, breathable, and suitable for wearable applications on the human body. The TED integrates an optical heartbeat sensor based on the PPG principle for respiration‐heartbeat synergistic monitoring, and data can be uploaded to an upper computer in real‐time via Bluetooth.

Based on the above theories and devices, this study innovatively designed a multi‐source fusion recognition system (Figure 1b) for specific scenarios like tumbles. The system constructs an intelligent model by collecting TED signals (normal/abnormal respiration and speech‐related airflow) and heartbeat signals (resting, exercising, and tumble‐related abnormal heartbeats) synchronously. After multi‐source fusion, where combined A‐TEDS (abnormal TED signals) and A‐HBS (abnormal heartbeat signals) are marked as abnormal and others as normal, the fused data is input into a deep‐learning model. The model can effectively detect abnormal situations and identify fall status.

### 2.2 Device and Performance Testing

**Figure 2:**
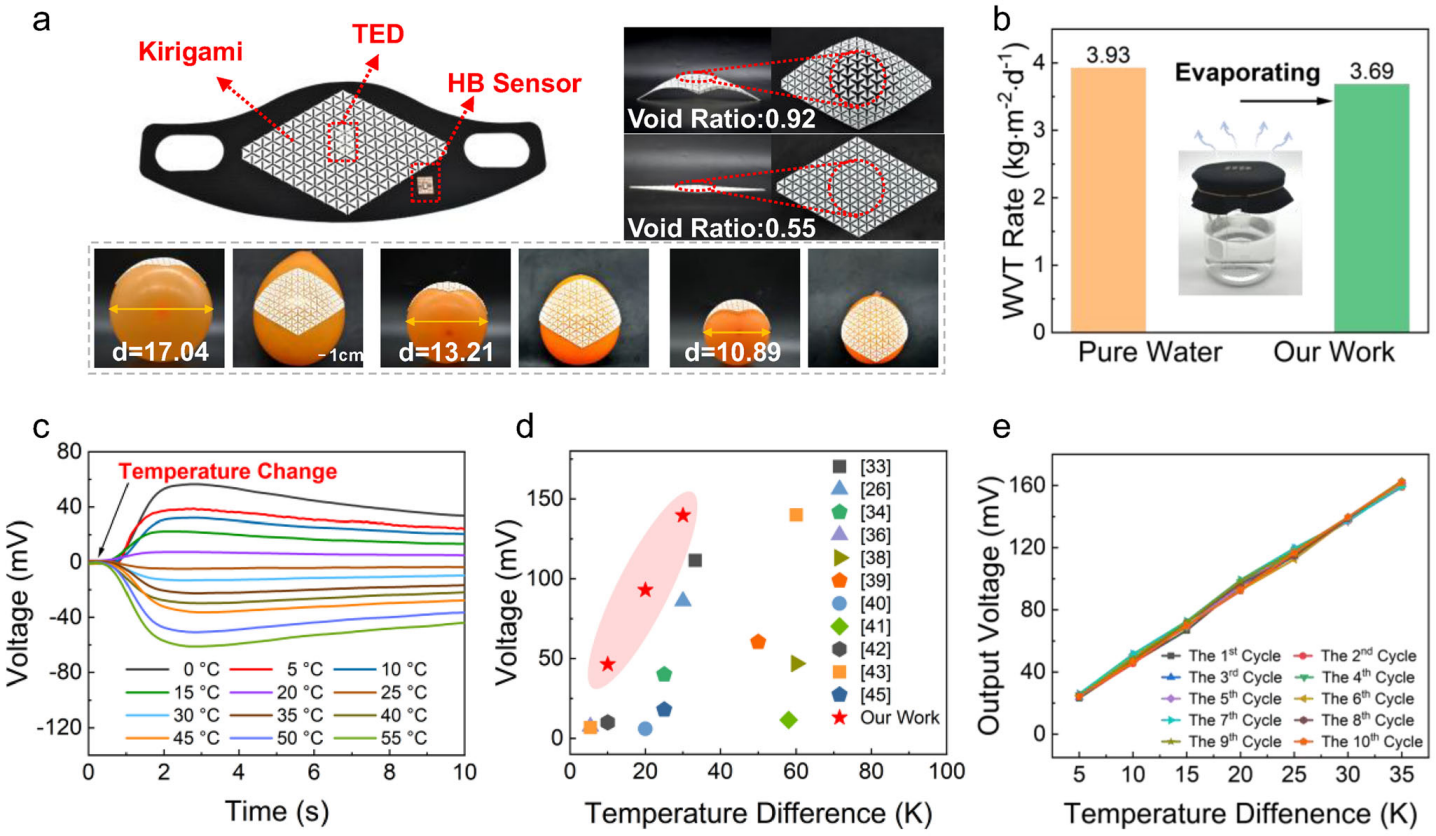
The device's performance test. a) The device and Kirigami properties. b) The WVT rate with and without the device obstruction. c) The output voltage curves of TED at different temperatures. d) A comparison of the output voltage with outstanding works in the past 5 years. e) The cyclic output voltage test.

The proposed system device consists of three parts (**Figure** **2**a), namely Kirigami, TED, and HB Sensor. Among them, Kirigami serves as the support layer of the mask to isolate the skin from the TED. This structure exhibits excellent stretchability, bendability, and out‐of‐plane deformability (Figure 2a, which can perfectly fit on balloons of different sizes), and the surface is smooth and flowing after deformation, suitable for comfortable wearing. Meanwhile, Kirigami provides superior breathability: its void ratio measures 0.55 in the undeformed state and increases to 0.92 near the TED after deformation, ensuring efficient airflow to the TED surface. To prove the breathability of the device, two beakers filled with 38 °C water (approximating human exhaled air temperature) were used to simulate respiratory conditions—one covered with the device and the other left uncovered. After 5 h, the weight difference was measured to calculate the water vapor transmission rate (WVT). The uncovered beaker showed a WVT of 3.93, while the covered one registered 3.69 (Figure 2b), confirming that the device maintains unimpeded vapor flow for wearer comfort and breathability (detailed calculations are provided in the Supporting Information).

Meanwhile, a series of performance characterizations were conducted on the TED. As shown in Figure 2c, the output voltage curve of the TE is tested by applying a temperature change to it in one direction, and the temperature interval is from 0 to 60 °C with an interval of 5 °C, and the curve shows that our device has excellent temperature output performance. As shown in Figure S2a (Supporting Information), the output voltage, current, and power density of the TE at temperature differences of 5, 10, 20, and 30 K were presented. The output voltage reached up to 139.7 mV, the output power is 2323.21 µW, and the output power density is 3704.1 µW cm^−2^ at a temperature difference of 30 K. A comparison of the output voltage (Figure 2d), power and power density (Figure S2b,c, Supporting Information) with similar works from the past 5 years.^[^ ^24^, ^26^, ^33^, ^34^, ^35^, ^36^, ^37^, ^38^, ^39^, ^40^, ^41^, ^42^, ^43^, ^44^, ^45^, ^46^, ^47^ ^]^ The cyclic output voltage test was shown in Figure 2e, which proves the stability. Meanwhile, finite element analysis in Figure S2d,e (Supporting Information) provides further evidence of the device's superior performance. The excellent robust mechanical properties of TEDs were shown in Figure S3 (Supporting Information). The portability of the TED was demonstrated through fabrication on masks comprising various materials, with details provided in Figure S4 (Supporting Information). The waterproof performance of the TED and its robustness against ambient humidity/temperature are presented in Figure S5 (Supporting Information).

### 2.3 Airflow‐Thermoelectric Response Theory and Airflow‐Speech Recognition

CHT is vital in daily life, e.g., the computer CPU heat dissipation, when a computer CPU overheats, the surrounding air heats up, causing its density to decrease and thus rise, while cooler air from the surroundings flows in to replace it. Air‐cooled cooling accelerates airflow over the CPU surface, enhancing heat transfer and improving CPU cooling efficiency. Similarly, when airflow of varying speeds passes over the surface of a TED, heat transfer occurs. Investigating the impact of this heat on the output of TEDs is crucial for broadening their application potential.

To explore the effect of airflow velocity on the response of TEDs, it is essential to first examine how airflow velocity influences the temperature of the upper surface of the TED. It is known from Newton's law of cooling:

(1) $$q_{conv}=hA\left(T_{air}-T_{upper}\right)=\frac{dQ_{conv}}{dt}$$

where*Q_conv_* is CHT, *A* is the surface area of the TED in contact with the fluid (m^2^), *T_upper_* is the temperature of the contact end of the TED with the fluid (K), *T_air_* is the fluid temperature (K), *h* is the coefficient of CHT, which is expressed in laminar convection over the surface of a flat TED as follows:

(2) $$h=\frac{\kappa Nu}{L}=\frac{0.664\kappa }{L}{\left(\frac{\rho_{air}\vec{\nu }}{\mu }\right)}^{0.5}Pr^{1/3}=h\left(\vec{\nu }\right)$$

where κ is the fluid thermal conductivity, *L* is the characteristic length, *Nu* is the Nussle number, ρ_*air*_ is the fluid density, μ is the hydrodynamic viscosity, *Pr* is the Prandtl number, and $\vec{\nu }$ is the fluid velocity vector. According to the CHT Formula:

(3) $$q_{conv}=mc_{p}\frac{dT_{upper}\left(t\right)}{dt}$$

where *m* is the mass of the material at the fluid contact end of the TED. Bringing in Equation (1), it can be obtained:

(4) $$\frac{dT_{upper}\left(t\right)}{dt}=\frac{h\left(\vec{\nu }\right)A}{mc_{p}}\left(T_{air}-T_{upper}\left(t\right)\right)=f\left(\vec{\nu }\right)\left(T_{air}-T_{upper}\left(t\right)\right)$$

Let the lower surface temperature of the TED be *T_lower_*. After air flow, the new upper and lower surface temperature difference is Δ*T_TED_*:

(5) $$\Delta T_{TED}=T_{upper}\left(t\right)-T_{lower}$$

The output voltage *V* of the TED is proportional to the temperature difference, which can be obtained by solving the differential equation:

(6) $$V=\alpha \left|\Delta T_{TED}\right|=\alpha \left|T_{air}-Ce^{-f\left(\vec{\nu }\right)\cdot t}-T_{lower}\right|$$

(7) $$C=T_{air}-T_{upper}\left(0\right)$$

*C* is a constant determined by the initial conditions. The detailed derivation process is provided in the Supporting Information.

**Figure 3:**
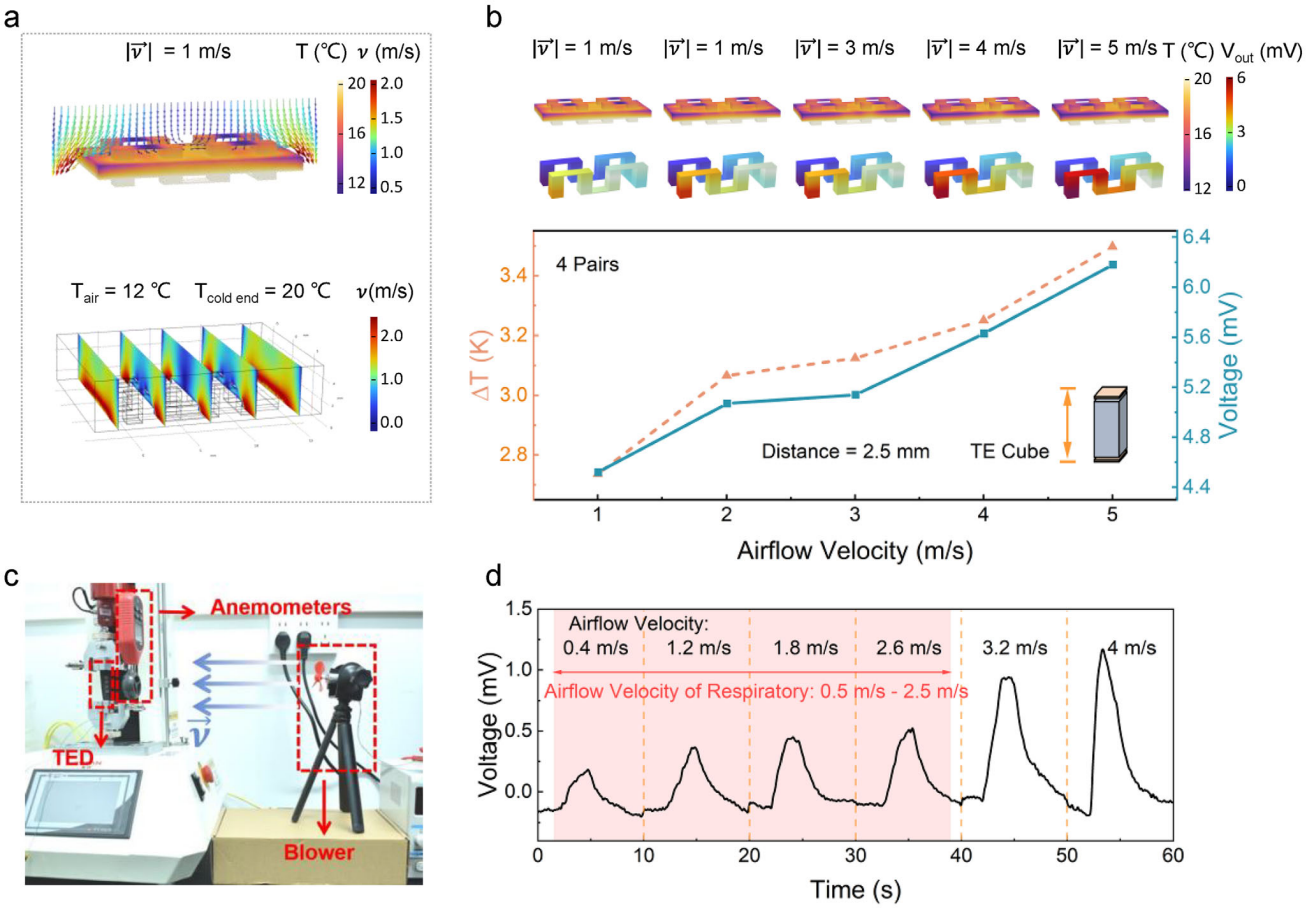
The simulation and experiment of airflow‐thermoelectric response theory. a) Temperature and fluid distribution, fluid velocity field section, and electric potential distribution. b) Variation of the TE body temperature difference and electric potential with the change of airflow velocity. c) The airflow velocity measurement setup. d) The output voltage of TED at different airflow velocities.

Simulations were performed based on this theory. **Figure** **3**a establishes a fluid‐solid coupled heat transfer field, with the governing equations for the thermoelectric temperature field as follows:

(8) $$\nabla \left(\kappa_{TED}\nabla T\right)+\rho_{TED}{\vec{j}}^{2}-T\vec{j}\left[\left(\frac{\partial \alpha }{\partial T}\right)\nabla T+\left(\nabla \alpha \right)T\right]=0$$

(9) $$\nabla \cdot \vec{j}=0$$

(10) $$\vec{j}=-\sigma \left(\nabla E+\alpha \nabla T\right)$$

where κ_*TED*_ is the thermal conductivity of TED, $\vec{j}$ is the current density, ρ_*TED*_ is the resistivity, α and *E* are the Seebeck coefficient and electrostatic potential, respectively. Equation (8) represents the conservation of energy, Equation (9) is the current continuity equation representing the conservation of charge, and Equation (10) is the generalized Ohm's law equation reflecting the coupling of electricity and heat in the thermoelectric effect. The governing equations for the fluid field under radiation‐free, steady‐state conditions are as follows:

(11) $$\rho_{Flow}\left(\vec{\nu }\cdot \nabla \right)\vec{\nu }=\nabla \cdot \left[-PI+K\right]+F$$

(12) $$\nabla \cdot \left(\rho_{Flow}\vec{\nu }\right)=0$$

where ρ_*Flow*_ is the fluid density, $\vec{\nu }$ is the fluid velocity vector, *P* is the pressure, *I* is the unit tensor, *K* is the viscous stress tensor, and *F* is the external force acting on the fluid. Equation (11) is theNavier‐Stokes equation showing the conservation of dynamics for a viscous incompressible fluid; in most common scenarios, air can be approximated as a viscous incompressible fluid, and this setting is followed here. Equation (12) is the continuity equation, which shows the conservation of mass of the fluid under radiation‐free, steady‐state conditions.

The boundary conditions of the simulation physical field are set as follows: 1) assuming that the airflow flows vertically to the upper surface of the TED. The inlet is the upper surface of the fluid field, and the outlet is the lower surface of the fluid field. Figure 3a shows a non‐uniform velocity distribution due to edge geometry effects. According to Bernoulli's principle, the reduced cross‐sectional area at thermoelectric device edges causes local flow acceleration. This expected phenomenon does not affect the results' validity. 2) The fluid temperature was set to 12 °C, and the lower surface temperature of the TED was set to a constant 20 °C. In this research, the average flow velocity at the inlet of the fluid field was varied from 1 to 5 m s^−1^. The outlet was set to have a pressure of 0; the other surfaces of the TED were set to be thermally insulated. And radiant heat is not considered. The parameters used in the simulation are shown in **Table** **1**.

**Table 1:** The parameters of the simulation.

Category	Seebeck Coefficient	Electric Conductivity	Thermal Conductivity
P‐type TE cube	202.5 × 10^−6^ V/K	9.25 × 10^5^ S/m	0.927 W/(m · K)
N‐type TE cube	−210 × 10^−6^ V/K	10 × 10^5^ S/m	0.965 W/(m · K)
Cloth electrode	/	/	0.3 W/(m · K)
Air	/	/	0.0257 W/(m · K)

When the inlet flow velocity was gradually increased from 1 to 5 m s^−1^ in 1 m s^−1^ increments, the temperature difference between the thermoelectric legs showed an increasing trend. Meanwhile, the potential distribution (Figure 3b) indicated that the output voltage rose with the increase in flow velocity, which is consistent with the theoretical expectation.

In this part, the effect of airflow velocity on the TE output voltage was experimentally investigated to validate the proposed theory. First of all, a testing platform was established, as shown in Figure 3c. The TED was clamped with a jig while an anemometer (UNI‐T363) was positioned directly in front. The blower, assembled with a servomotor and fan blades, was placed on a stand at the same horizontal level as the TED and the anemometer. The airflow velocity of the blower was controlled by a DC power supply and was used to blow onto the surface of the TED. The voltage generated by the action of the airflow on the TED was collected by a National Instruments (NI) data acquisition card. Figure S6a–c (Supporting Information) tests the response time of the TED; our response speed is relatively fast and ranks among the top in similar research.^[^ ^33^, ^48^, ^49^, ^50^ ^]^ The output voltage of the TED at airflow velocities ranging from 0.5 to 4 m s^−1^ was further measured (Figure 3d). It can be observed that as the airflow velocity increases, more heat exchange occurs between the air and the surface of the TED, establishing a larger temperature gradient. Consequently, this leads to a higher output voltage of the TED, validating our theory once again. Human respiratory airflow velocity was measured, which falls within the range of 0.5 to 2.5 m s^−1^ (Figure S6d, Supporting Information), therefore, the device is fully capable of meeting the requirements for respiratory detection. In addition, Figure S6e,f (Supporting Information) demonstrates that the proposed device maintained stable performance during long‐term wear, meeting the daily requirements for wearable applications.

**Figure 4:**
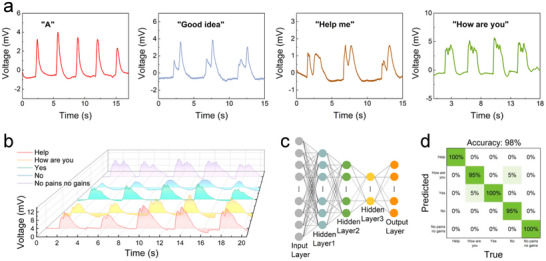
Application of the TED in speech recognition. a) Voltage waveforms for the speeches of “A”, “Good idea”, “Help me”, and “How are you”. b) Voltage spectrum for the five speeches. c) Structure diagram of the FCNN. d) Confusion matrix for the recognition results of the speech “HELP”.

Inspired by previous research, since different speeches often have distinct respiratory airflow, the potential of this device in speech recognition was explored. As shown in **Figure** **4**a, experiments were conducted on speeches such as “A”, “help me”, “good idea”, and “how are you” (Voltage diagrams corresponding to more speeches are provided in Figure S7, Supporting Information, for reference). It can be observed that each speech exhibits a unique, clear, and reproducible voltage waveform. These results demonstrate that the TED has considerable application value in speech recognition. However, in medical emergencies, recognizing distress speeches is of crucial importance. Therefore, this work focused on distress speeches like “help” and some common speeches such as “YES”, “no”, “no pains no gains”, and “How are you”, and presented their voltage spectra in Figure 4b. Ten samples were collected for each speech, with 9 samples used for training and 1 sample for testing. Figure 4c shows a fully‐connected neural network (FCNN) model^[^ ^51^ ^]^ constructed for speech recognition. Figure 4d indicates that the recognition accuracy of this model is as high as 98%, and the loss function is presented in Figure S8 (Supporting Information).

### 2.4 Practical Applications under the Theory

**Figure 5:**
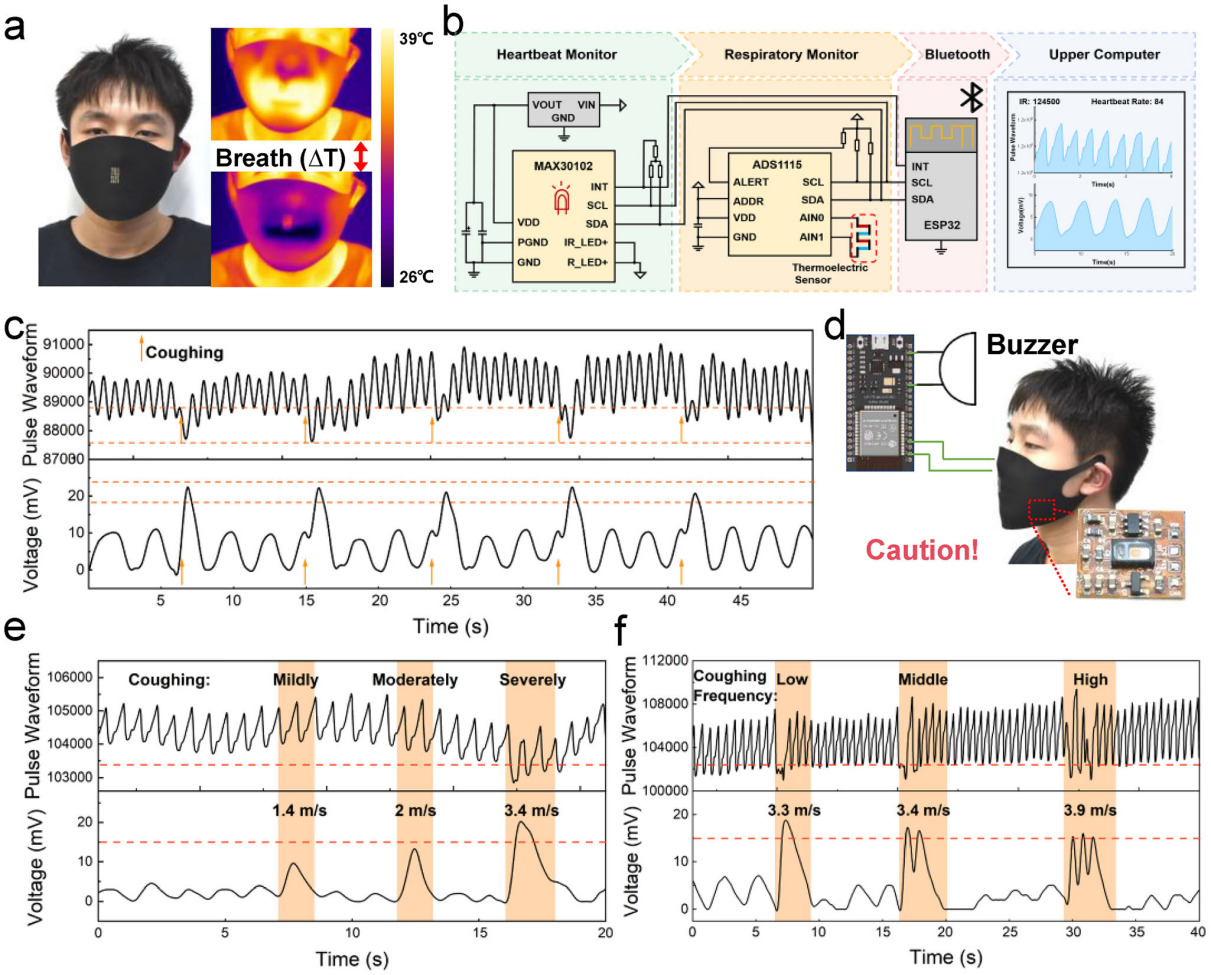
System circuit and its cough alarm application. a) Respiratory imaging under infrared. b) System circuit diagram. c) Respiration and heartbeat waveform under coughing state. d) The alarm system diagram. e) Respiration and heartbeat waveform under different cough intensities. f) Respiration and heartbeat waveform under different frequencies of severe coughing.

To demonstrate the practical value of this research, the system was used for respiratory and heartbeat monitoring. **Figure** **5**a shows the infrared images of exhalation and inhalation, from which a significant temperature difference during the breathing process can be observed. When exhaling, the gas temperature is much higher than the environment temperature (the dark part in the figure). The increased heat exchange between the hot airflow and the surface of the TED causes the TED output voltage to rise. When inhaling, the CHT removes the heat from exhalation, resulting in a decrease in the output voltage, which verifies that our device can detect respiration. The TED integrated with an optical sensor to form a wearable system capable of synergistically monitoring respiration and heartbeat. Figure 5b presents the circuit diagram of the system. Heartbeat and data can be uploaded to the upper computer in real‐time for dynamic monitoring. The heartbeat and respiration during sitting, walking, and running were tested. Different waveforms and frequencies were shown under different exercise states, indicating that our device has excellent motion‐recognition ability and can stably capture signals under different exercise states (Figure S9, Supporting Information).

To further prove the practical value of our device in health monitoring, cough tests were conducted using this system. The waveforms in Figure 5c are the respiratory voltage waveform and the heartbeat waveform during a volunteer's cough. It is characterized by a significant increase in the voltage peak and a decrease in the heartbeat intensity. Based on this, an alarm system was developed to track the wearer's cough status and trigger a buzzer alarm when a cough is detected (Figure 5d). In the design of the alarm system, the respiration‐heartbeat waveforms under different cough intensities were first tested (Figure 5e) and severe cough was selected as a necessary condition to trigger the alarm. Then, the frequency of severe coughs was tested (Figure 5f). When the number of coughs is greater than or equal to three within a 5 s time interval, the alarm will be triggered.

### 2.5 Multi‐Source Fusion Recognition

Single distress speeches/physiological parameters have limitations in healthcare. On the one hand, a single distress speech is susceptible to environmental interference and user physiological limitations; on the other hand, independent heartbeat or respiration parameters are deceptive for abnormal condition detection. This limitation is especially prominent in emergency healthcare scenarios, where users experiencing cardiac arrest or accidental falls typically exhibit characteristic multi‐parameter synergistic variations‐potentially manifesting both active distress through speeches and passive symptoms including weakened respiration and irregular heart rate. Therefore, the proposed system employs deep learning technology to enable multi‐source fusion recognition of speeches, breaths, and heartbeats (Figure S10, Supporting Information), which helps to provide users with rapid response and judgment in critical situations.

**Figure 6:**
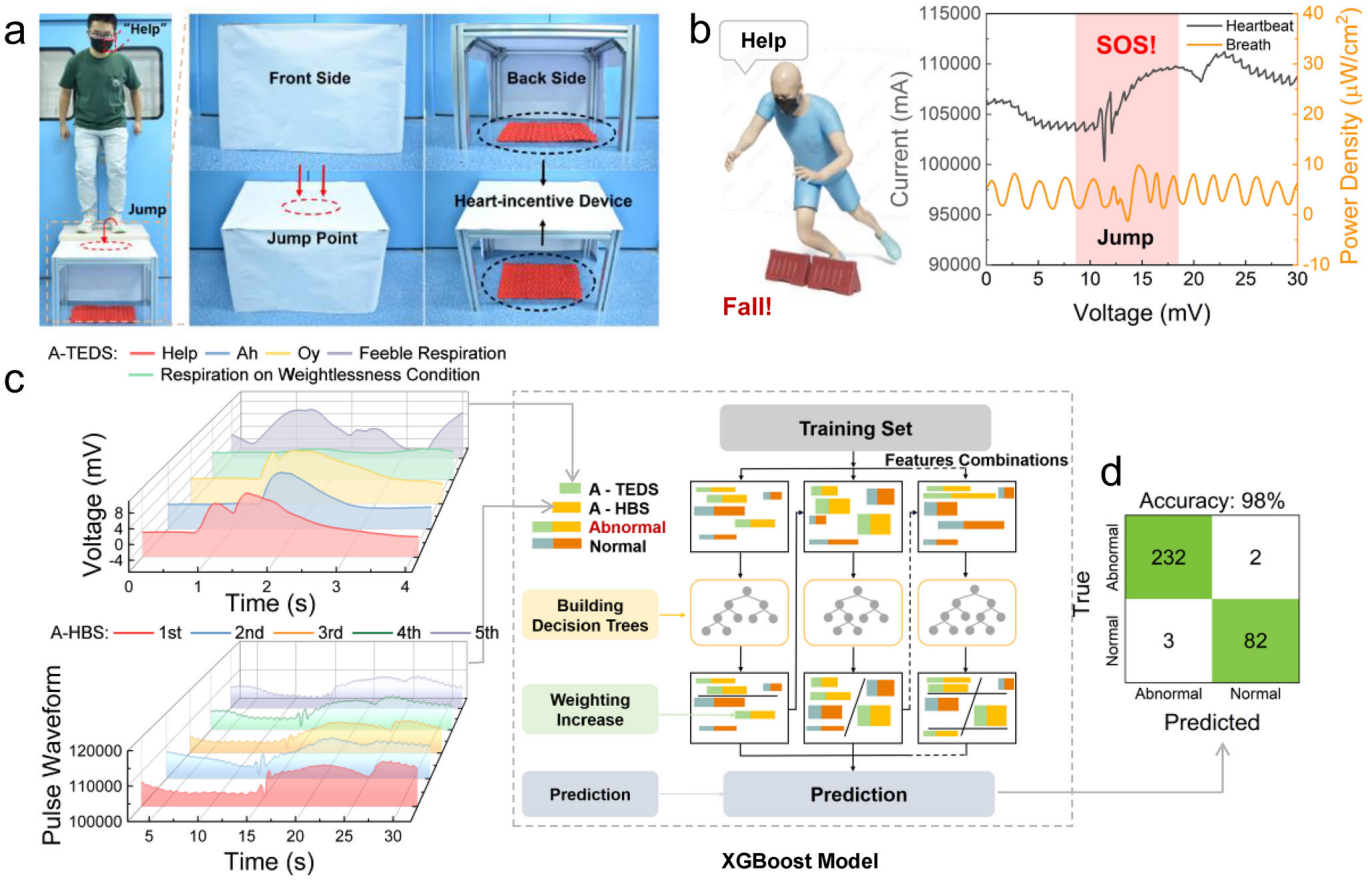
Multi‐source fusion recognition for critical scenario. a) The abnormal heartbeat collection device. b) Abnormal heartbeat and weightlessness respiration waveform during collection. c) Multi‐source fusion recognition framework for A‐TEDS and A‐HBS, on the left is the spectrum of A‐TEDS and A‐HBS, and on the right is the block diagram of the XGBoost model. d) Confusion matrix of the multi‐source fusion recognition.

First, this work simulated the abnormal heartbeat during a tumble by applying a pain stimulus in a weightless state. The collection device is shown in **Figure** **6**a. The wearer wears the mask and jumps onto the collection equipment while shouting “Help”, “Ah”, “Oy”, taking feeble respiration or breathing during weightlessness (without shouting any speeches) (abnormal respiration), which are defined as A‐TEDS to fully simulate the actual scenario. Each phrase has 7 sets of data, which were derived from three different volunteers respectively. Feeble respiration was simulated by adding a breathing resistor. The collection equipment was built with a 40 cm high steel frame. The top and front of the frame were covered with white paper to ensure that the volunteers were unaware of what was under the frame. Under the frame, a heart‐incentive device or sponge to increase randomness was placed. The pain‐stimulating heartbeat generated by the heart‐incentive device was regarded as an abnormal heartbeat (A‐HBS). Meanwhile, the steel frame was lowered to 20 cm and placed nothing under it to create a micro weightlessness state. To distinguish from the weightless state, the heartbeat when jumping from it was defined as a normal heartbeat. The waveform diagrams of weightlessness respiration and abnormal heartbeat are shown in Figure 6b. Figure 6c shows the framework of deep learning. On the left are the spectra of A‐HBS and A‐TEDS. Given the huge amount of data, which may impose a heavy burden on the neural network, the faster XGBoost algorithm was selected (Table S1, Supporting Information). Multi‐source fusion was performed on the A‐HBS and A‐TEDS data as the input dataset for the XGBoost model (on the right of Figure 6c). The combination of AHBS and A‐TEDS was defined as “abnormal”, while the other three cases: the combination of normal speeches and A‐HBS, the combination of normal speeches and normal heartbeats, and the combination of A‐TEDS and normal heartbeats were defined as “normal”. As shown in Figure 6d, the accuracy of the recognition results reached 98%, which proves the reliability of the adopted methodology.

## 3 Conclusion

This work developed a novel fabric‐based TE wearable system with superior flexibility and TE properties, where a kirigami was introduced as a support layer to prevent direct skin‐TED contact and ensure precise sensing, while conforming seamlessly to curved surfaces. This system enables wireless real‐time monitoring of respiratory and heartbeat signals for medical applications. In this paper, the scientific correlation between airflow velocity, heat, and the output voltage of TEDs is deeply investigated, which strongly confirms the feasibility of TEDs applied to airflow detection. Notably, based on this innovatively established theory, we pioneered the application of thermoelectric devices in speech recognition. Furthermore, by integrating optical sensors and deep learning algorithms, we achieved multi‐source recognition of respiration, speech, and heartbeat with 98% accuracy in identifying critical scenarios such as fall detection, significantly improving the reliability of single‐parameter medical monitoring. The successful implementation of a cough alarm further demonstrates the practical potential of this system in healthcare applications. This research not only advances the field of wearable medical devices but also provides a foundation for future innovations in intelligent health monitoring technologies.

## 4 Experimental Section

### Materials

Bi_0.5_Sb_1.5_Te_3_ (p‐type) and Bi_2_Te_2.7_Se_0.3_ (n‐type) TE legs were purchased from Hubei Sagreon New Energy Technology Company, Ltd., and the cuboids was custom‐made with the size of 1.4 mm × 1.4 mm × 2.5 mm, the thermoelectric properties of the material can be found in Table 1. The fabric electrodes (total thickness with the paper substrate: ≈130 µm, without the substrate: ≈70 µm, conductive component: electroplated Cu‐Ni alloy) were purchased from Shenzhen Shunzhi Technology.

### Fabrication of the TED

First, the fabric substrate was laser‐cut to create apertures that perfectly accommodated the TE cubes. Simultaneously, we laser‐cut rectangular fabric electrodes, which were designed to enhance flexibility while ensuring their high performance, avoiding the high resistance of serpentine electrodes. Subsequently, the distribution gaps for the TE cubes were cut on a steel mesh. These gaps were then utilized to position the fabric electrodes, which were secured in place with heat‐release adhesive. Then, solder paste was applied to the fabric electrodes. The TE cubes were then inserted through the steel mesh in an alternating P‐N‐P sequence and were precisely positioned into the solder‐coated fabric electrodes. The assembly was heated on a 180 °C hotplate for 10 min. After that, the soldered assembly was placed on a flat surface, the steel mesh was removed, and the fabric substrate was aligned and pressed down, causing the TE cubes to be accurately embedded into the fabric substrate. The bottom electrodes were then assembled using the same technique. Finally, a 120 °C hot gun was used to heat the heat‐release adhesive, allowing it to release, and thus the fabrication of the respiratory module was completed.

After the Kirigami structure was designed using SolidWorks software, it was printed with a fused deposition modeling (FDM) 3D printer. Upon completion of printing, the mask was folded, and the two ends of the folded mask were positioned. The mask was then unfolded, and the Kirigami structure was placed on the mask for alignment. Finally, sewing technology was used to accurately sew the Kirigami layer onto the mask. Detailed steps are shown in Figure S11 (Supporting Information). Details of the Kirigami structure design are available in Figure S12 (Supporting Information).

### Characterization

The output performances of the TED were measured by a Keithley 2400 source meter. The internal resistance of the TED was measured using an electrochemical analyzer (CHI650e, CH Instruments, Inc.). The infrared image was captured by an infrared camera (Ti480 PRO FLUKE). The temperature difference between the thermoelectric devices was measured by the thermocouple (JK‐8A).

## Conflict of Interest

The authors declare no conflict of interest.

## Supporting information



Supporting Information

Supplemental Movie 1

## Data Availability

The data that support the findings of this study are available in the supplementary material of this article.
