# Supplementary material for: Breath, Pulse, and Speech: A Multi‐Parameter Wearable System using Airflow‐Thermoelectric Fusion Technology
Source: Adv Sci (Weinh). 2025 Oct 27;13(4):e14277. doi: 10.1002/advs.202514277 (PMC12822444; doi:10.1002/advs.202514277)
Supplement: Supplementary file 1 — Supporting Information [file ADVS-13-e14277-s002.docx]

Supporting Information

Breath, Pulse, and Speech: A Multi-Parameter Wearable System Using Airflow-Thermoelectric Fusion Technology

Zheng Zhu, Xinxin Yan, Yue Hou*, Yuxuan Zeng, Zhaoyu Li, Xiaolong Sun, Chang Li, Xiaosa Liang, Qianfeng Ding, Ziyu Wang* and Cheng Lei*

This supplement contains:

Supplementary Figures 1-10

Supplementary Table 1

**Airflow-thermoelectric response theory:**

According to the heat balance equation, this relationship can be systematically analyzed. According to the heat balance equation (Equation. (1)):

| $\rho c_{p}\frac{\partial T}{\partial t}=Q_{Fourier}+Q_{Joule}-Q_{conv}$ | (1) |
| --- | --- |

Where $\rho$ is the density of the material at the end of the TED in contact with the fluid, $c_{p}$ is the specific heat capacity of the material, $Q_{Fourier}$ is Fourier heat, $Q_{Joule}$ is Joule heat, $Q_{conv}$ is CHT. Among them, CHT is related to the airflow velocity, so focus on the discussion. It is known from Newton's law of cooling:

| $q_{conv}=hA\left( T_{air}-T_{upper} \right)=\frac{dQ_{conv}}{dt}$ | (2) |
| --- | --- |

Where $A$ is the surface area of the TED in contact with the fluid (m^2^), $T_{upper}$ is the temperature of the contact end of the TED with the fluid (K), $T_{air}$ is the fluid temperature (K), $h$ is the coefficient of CHT which is expressed in laminar convection over the surface of a flat TED as follows:

| $h=\frac{\kappa Nu}{L}=\frac{0.664\kappa}{L}(\frac{\rho_{air}\vec{\nu}}{\mu})^{0.5}{Pr}^{1/3}=h(\vec{\nu})$ | (3) |
| --- | --- |

Where $\kappa$ is the fluid thermal conductivity, *L* is the characteristic length, $Nu$ is Nussle number, $\rho_{air}$ is the fluid density, $\mu$ is the hydrodynamic viscosity, $Pr$ is the Platt number, and $\vec{\nu}$ is the fluid velocity vector. Thus, the CHT capacity can be expressed as:

| $q_{conv}=h(\vec{\nu})A(T_{air}-T_{upper}(t))$ | (4) |
| --- | --- |

According to the CHT Formula:

| $q_{conv}=mc_{p}\frac{dT_{upper}(t)}{dt}$ | (5) |
| --- | --- |

Where $m$ is the mass of the material at the fluid contact end of the TED. Bringing in Equation (4), it can be obtained:

| $\frac{dT_{upper}(t)}{dt}=\frac{h\left( \vec{\nu} \right)A}{mc_{p}}\left( T_{air}-T_{upper}(t) \right)=f(\vec{\nu})\left( T_{air}-T_{upper}(t) \right)$ | (6) |
| --- | --- |

It can be obtained by solving the differential equation:

| $T_{upper}\left( t \right)=T_{air}-Ce^{-f\left( \vec{\nu} \right)\cdot t}$ | (7) |
| --- | --- |
| $C=T_{air}-T_{upper}(0)$ | (8) |

$C$ is a constant determined by the initial conditions. Let the lower surface temperature of the TED be $T_{lower}$. After air flow, the new upper and lower surface temperature difference is $\Delta T_{TED}$:

| $\Delta T_{TED}=T_{upper}\left( t \right)-T_{lower}$ | (9) |
| --- | --- |

The output voltage $V$ of the TED is proportional to the temperature difference, which can be obtained by substitution:

| $V=\alpha\left\vert\Delta T_{TED} \right\vert=\alpha\left\vert T_{air}-Ce^{-f\left( \vec{\nu} \right)\cdot t}-T_{lower} \right\vert$ | (10) |
| --- | --- |

**Water vapor transmission rate calculation:**

Breathability is a necessary quality for wearable devices intended for long-term use, as it increases the wearer's comfort and reduces the incidence of skin inflammation. While maintaining the dryness and stability inside the device, it also adapts to different environments. To test the breathability of the TED, we prepared two beakers containing water at 38°C, which is closest to the temperature of human breath for simulating the breathability of respiration, one covered with the TED and the other left uncovered. After 5 hours, we compared the weight changes before and after to calculate the Water Vapor Transmission (WVT) rate.

|  | $WVT=\frac{m_{1}-m_{2}}{S}\times24$ | (1) |
| --- | --- | --- |

$m$ represents the weight of the beaker before the experiment, and m_2_ is the weight after the experiment. S denotes the effective experimental area.


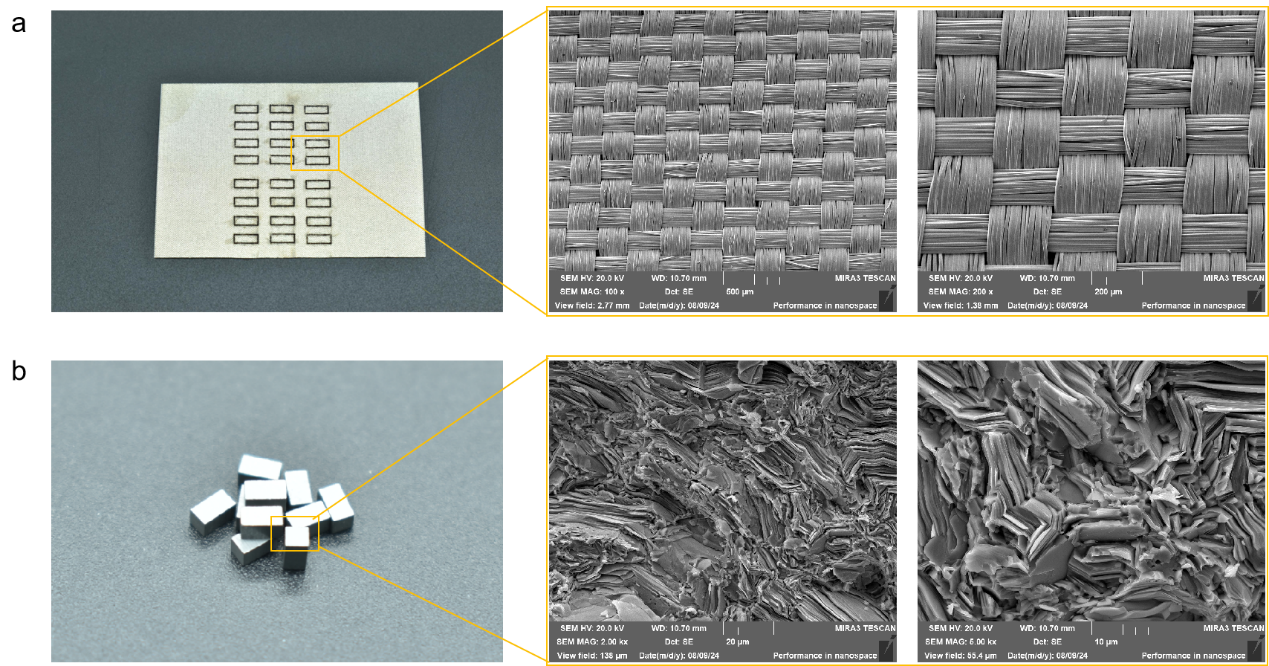


Figure S1. The scanning electron microscope (SEM) on a) the cloth electrodes and b) the TE cubes

**Figure S1a** shows the cloth electrode, the SEM and magnified on cloth electrode. Figure. S1b is a TE cubes, the SEM and magnified on TE cubes.


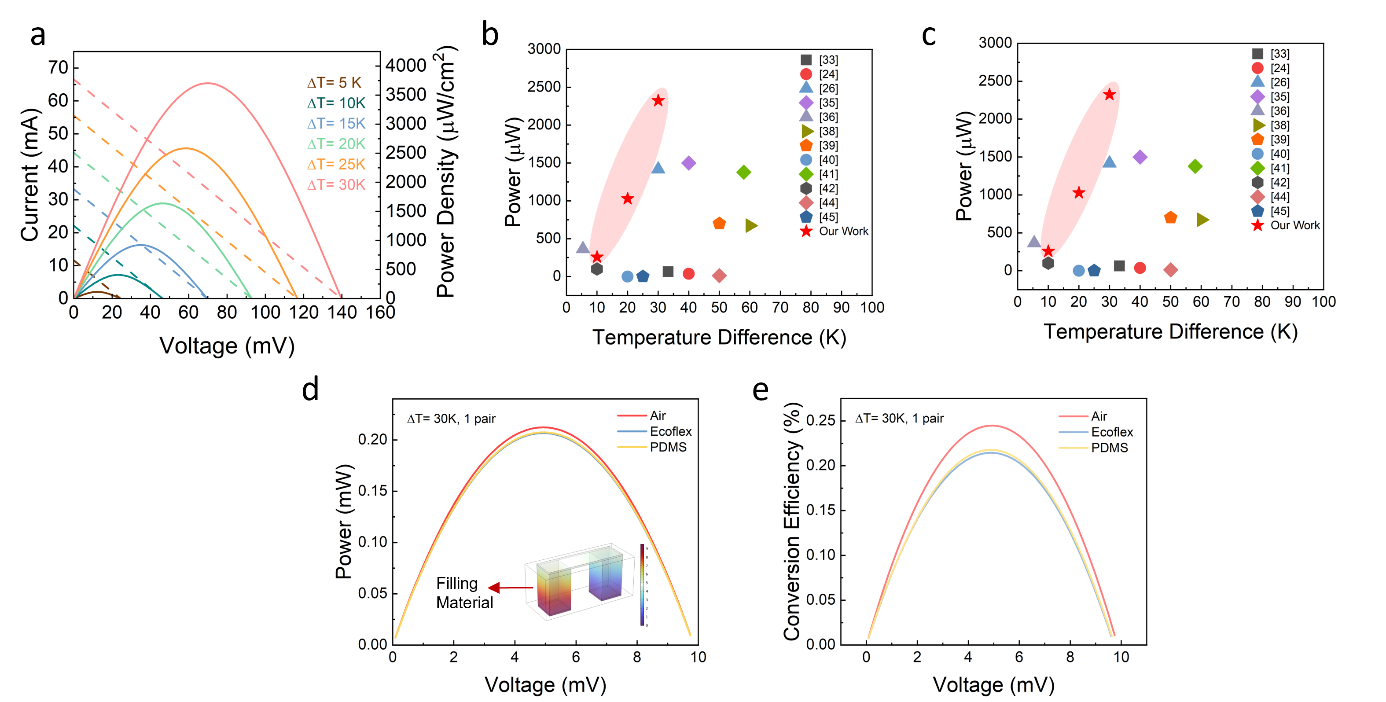


Figure S2. A comparison of the a) the ouput performance of the TED. b) voltage and c) power with outstanding works in the past 5 years. Finite element analysis of d) output power and e) conversion efficiency with air, Ecoflex, PDMS Filling Materials.

The output voltage, current and power density of TE at temperature difference of 5 K, 10 K, 20 K and 30 K, the output voltage is as high as 139.7mV, the output power is 2323.21 uW, and the output power density is 3704.1uW/cm2 at a temperature difference of 30K. We compared power and power density with outstanding works of last 5 years in Figure S2b and Figure S2c. These works encompass thin-film-based TED, textile-based TED and cubes-based TED.

The temperature difference between the cold end and the hot end of the TE cubes directly affects the output performance of TEDs. The larger the temperature difference is, the higher the output power will be. Therefore, how to ensure the temperature difference between the two ends has become an optimization scheme for researchers. Existing works usually adopt Ecoflex and PDMS as filling layer material, which possess low thermal conductivity and are soft. However, in our work, air is chosen as the filling material. The thermal conductivity of air is even lower than that of Ecoflex and PDMS, which can better reduce heat leakage and establish a larger temperature difference, resulting in higher output power.

We verify the superiority of our work through finite element analysis. We compared the effects of different filling layer materials, such as air (in our work), Ecoflex, and PDMS, on the thermoelectric performance. As shown in the Figure S2c, under the condition of an externally applied temperature difference of 30 K, for the work with air as the filling material (one pair of TE cubes), the output power is 209 μW, while the output power of the work with Ecoflex and PDMS as filling materials does not exceed 203 μW. Additionally, Figure S2d compares the effects of the three materials on the conversion efficiency of the TED. It can be seen that the conversion efficiency of the TED with air as the filling material reaches 2.4%, while the conversion efficiencies of Ecoflex and PDMS are only around 2.1%. This difference contributes to improving the output performance of wearable TEDs. Thus, it is evident that our device has excellent output performance.


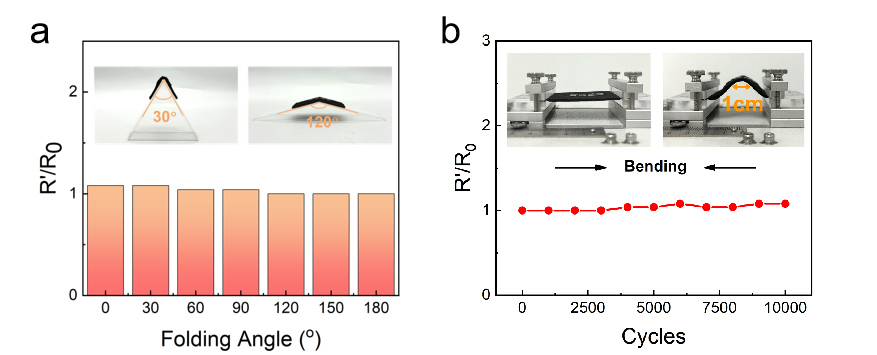


Figure S3. a) Folding performance of TED. b) Bending performance of TED.

As shown in Figure S3a, resistance was tested when TED was folded from 0° to 180º with step of 30º, which shows the resistance remained nearly consistent with that of the unfolded state. Furthermore, after subjecting it to 10,000 bending cycles, the resistance still showed minimal change (Figure S3b). This demonstrates that our device is well-suited for wearable applications.


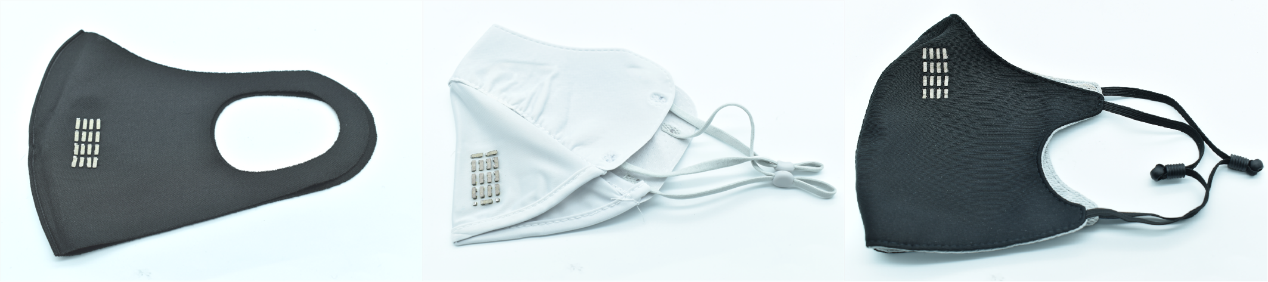


Figure S4. Weaving TEDs into masks made of various materials.

TEDs have been integrated into masks made from various materials, as shown in **Figure S4**, which from left to right are a knitted mask, a silk mask, and a cotton mask. It is evident that our TEDs possess a considerable portability, enabling them to be incorporated into a wide array of garments, demonstrating their promising wearable characteristics.


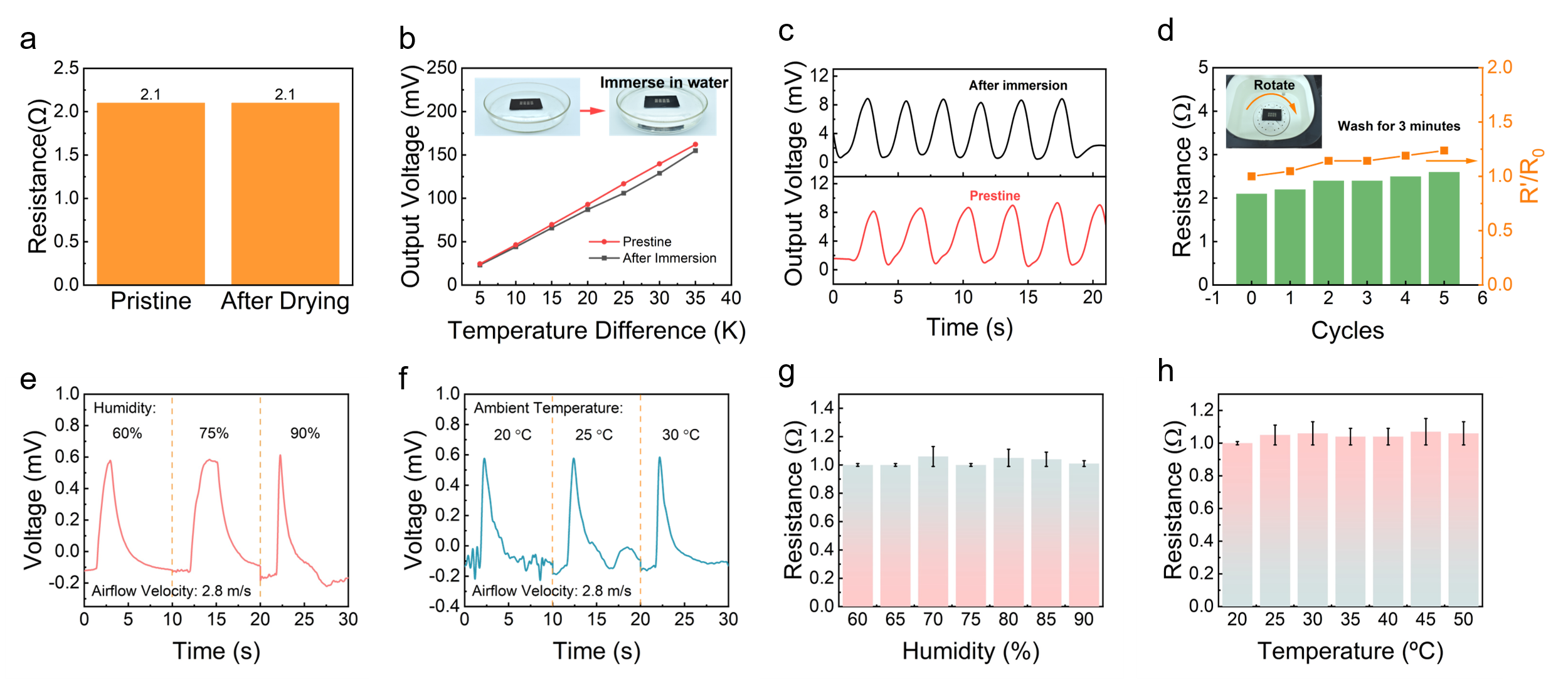


Figure S5. a) The resistance before and after immersion. b) The output voltage curve of TED before and after immersion. c) The respiratory waveform of TED before and after immersion. d) The resistance before and after washing. Output voltage waveform of the thermoelectric device (TED) at an airflow velocity of 2.8 m/s under e) different humidity conditions, and f) different temperature conditions. Internal resistance of the TED at an airflow velocity of 2.8 m/s under g) different humidity conditions, and h) different temperature conditions.

**Figure S5a-c** shows that the TED was immersed in water for 5 hours, and then dried in 50°C oven for 1 hour, and tested its resistance and output voltage before and after immersion.

Figure S5d illustrates the resistance change of the TED after being washed 5 times in a mini washing machine, with each wash cycle lasting 3 minutes, followed by 1 hour of drying in a 50°C oven. The resistance only slightly increased from 2.1 Ω to 2.6 Ω.

Figures S5e-f show the output voltage waveform and resistance of the TED when exposed to a 2.8 m/s airflow, with the ambient humidity increasing from 60% to 90%. Figures g–h present the output voltage waveform and resistance of the TED under a 2.8 m/s airflow after changes in ambient temperature. It can be observed that our device exhibits robustness against both humidity and temperature variations.


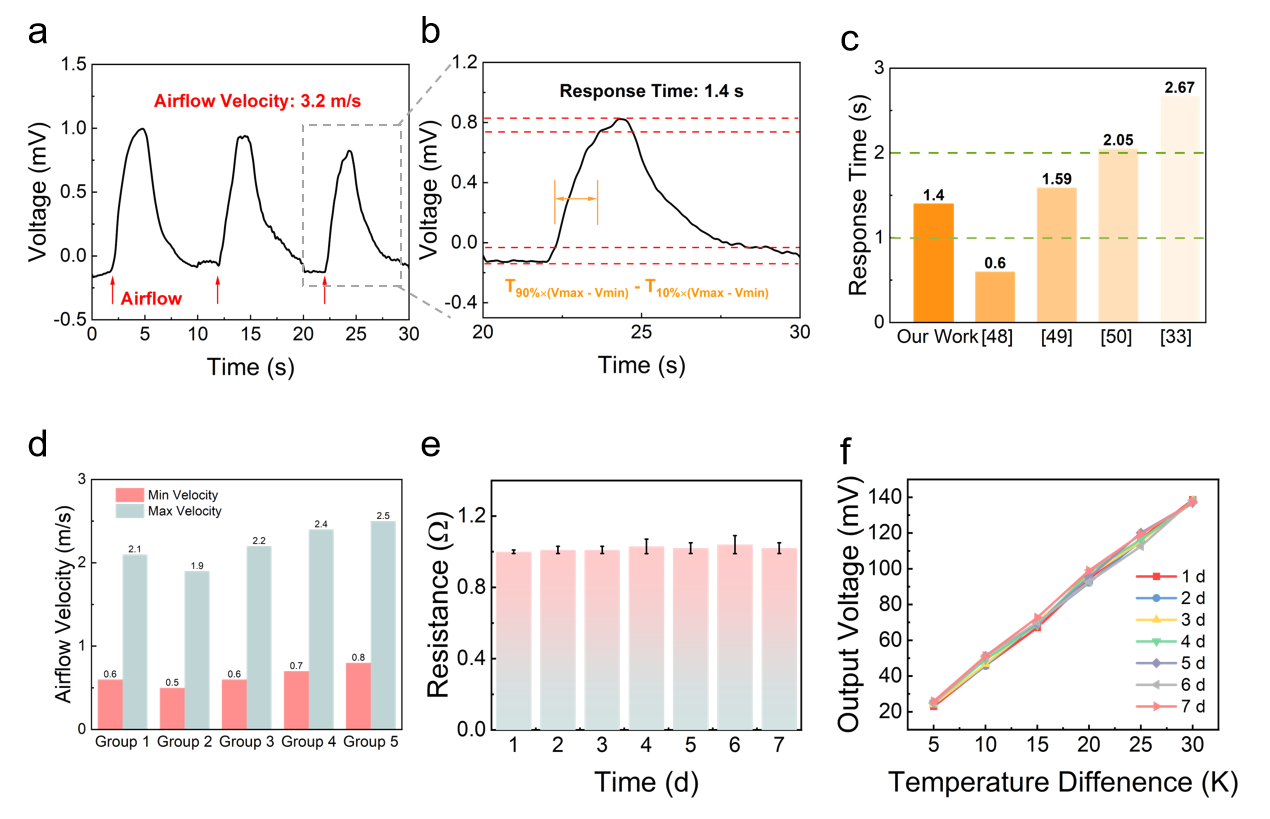


Figure S6. a) The output voltage graph of TED at an airflow velocity of 3.2 m/s, along with the response time curve of b). c) A comparison of our response velocity with other literature. d)Test of human respiratory airflow velocity range. e) Resistance and f) output voltage of the TED during a 7-day operation, with over 3 hours of working time per day.

Figure S6a shows the output voltage of the TED when the airflow velocity is 3.2 m/s and the environment temperature is 26 °C. Figure S6b is a partial enlargement of Figure S6a, in which the response time is calculated to be 1.4 s. When comparing our work with similar researches over the past five years (Figure S6c), our response speed is relatively fast and ranks among the top in similar research ^[33, 48-50]^.

We enlisted 5 volunteers to test the human respiratory airflow velocity range. The anemometer described in the manuscript was placed directly in front of the nose and contacted with the nose, which was used to match the distance between the mask and the nose, and the respiratory airflow velocity range of the 5 volunteers was tested separately, as shown in **Figure S6**. It can be seen that the human respiratory airflow velocity was in the range of 0.5 m/s to 2.5 m/s.

We conducted a durability test on the device, where a volunteer wore the device for 7 days with sensing operations lasting over 3 hours each day. As shown in Figure S6e, the resistance of the device during the 7-day period remained relatively stable with minimal fluctuations. Figure S6f presents the output voltage under different temperature differences, and the response trend of the output voltage remained consistent within 7 days. These results indicate that the device maintains stable performance during long-term wear.


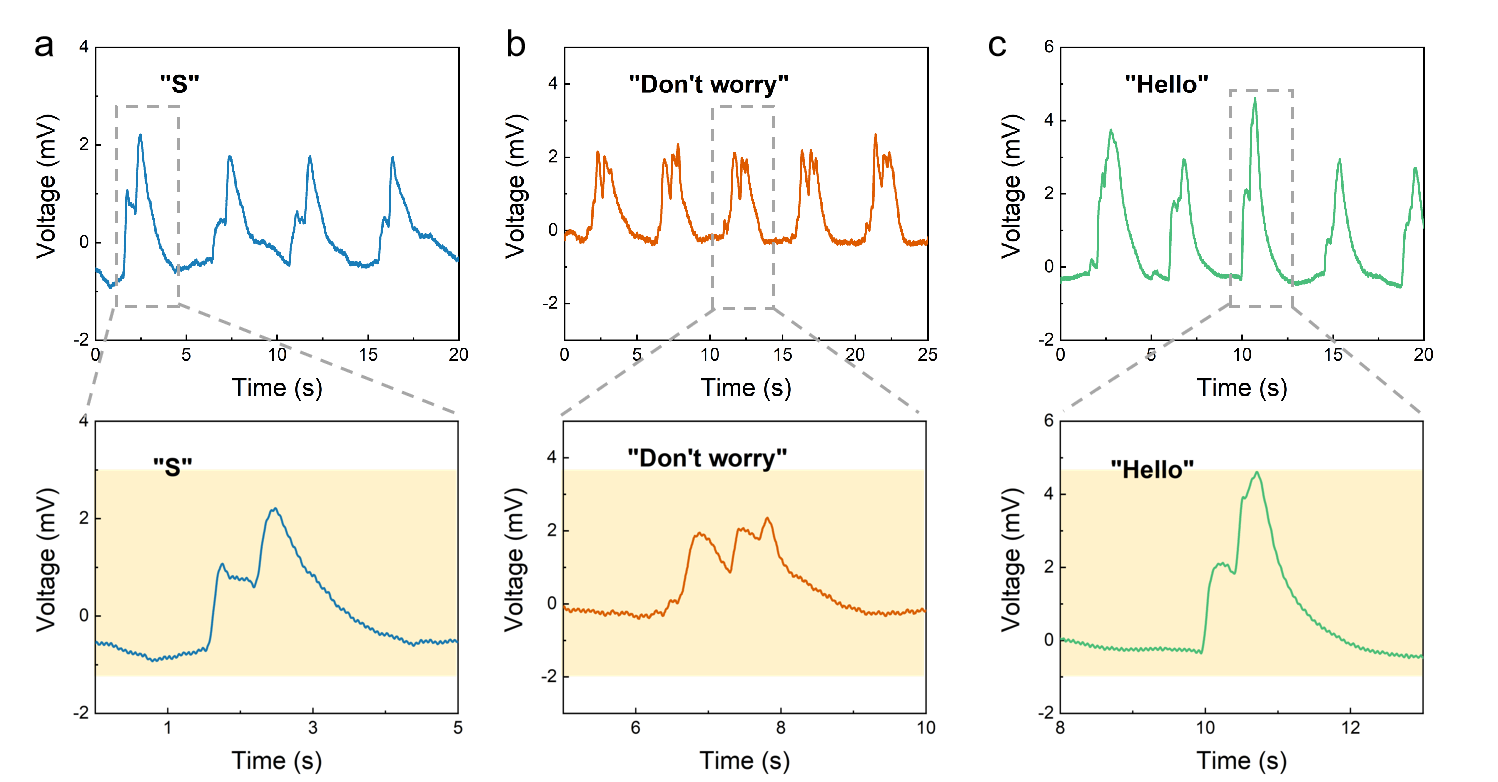


Figure S7. a-c) The phrase-voltage curves of “S”, “Don’t worry” and “Hello”.

**Figure S7** We tested the phrases “S”, “Don’t worry” and “Hello” using TED, and the different phrases are Different phrases have different characteristic waveforms and are clear and repeatable.


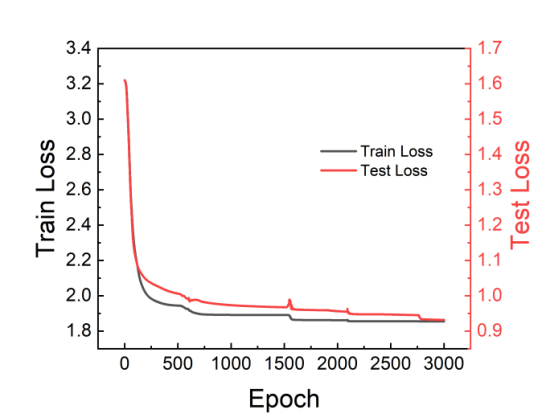


Figure S8. The loss function of FCNN.

We developed a FCNN model based on PyTorch. In this model, the input layer consists of 4096 neurons (corresponding to a sampling frequency of 0.001 Hz and a sampling time of 4.096 seconds). The hidden layer structure is 128*64*32, and the output layer corresponds to the number of text labels to be recognized, which is 5 in this case. Considering the advantage of ReLU in training time, we chose it as the activation function for the hidden layer neurons. We applied the Softmax activation function to the output neurons to map the probabilities of the labels to the range of 0 to 1. The loss function of the model is the cross-entropy loss function (**Figure S8**), defined as:

|  | $H\left( p,q \right)=-\sum_{i=1}^{n} p\left( x_{i} \right)\ln q(x_{i})$ | (2) |
| --- | --- | --- |

In the formula, *x_i_* represents the *i*-th sample, *p*(*x_i_*) is the true label of *x_i_* (often represented using one-hot encoding), and q(*x_i_*) is the predicted probabilities corresponding to different predicted labels.

In the experiment, we selected 5 phrases (including words) for recording: "yes," "no," "no pains no gains," "help," and "how are you," each recorded 10 times. To improve the generalization ability of the model, we introduced 9 additional instances of additive Gaussian noise (with a mean of 0 and a standard deviation of 1mV) to each initial waveform, thus achieving data augmentation. During the model training process, we employed the Adam optimizer with a batch size of 200 and an initial learning rate of 0.001. We applied learning rate decay when the training loss ceased to converge. After 3000 epochs of training, both the training and test losses showed significant convergence, and the model achieved impressive accuracy.


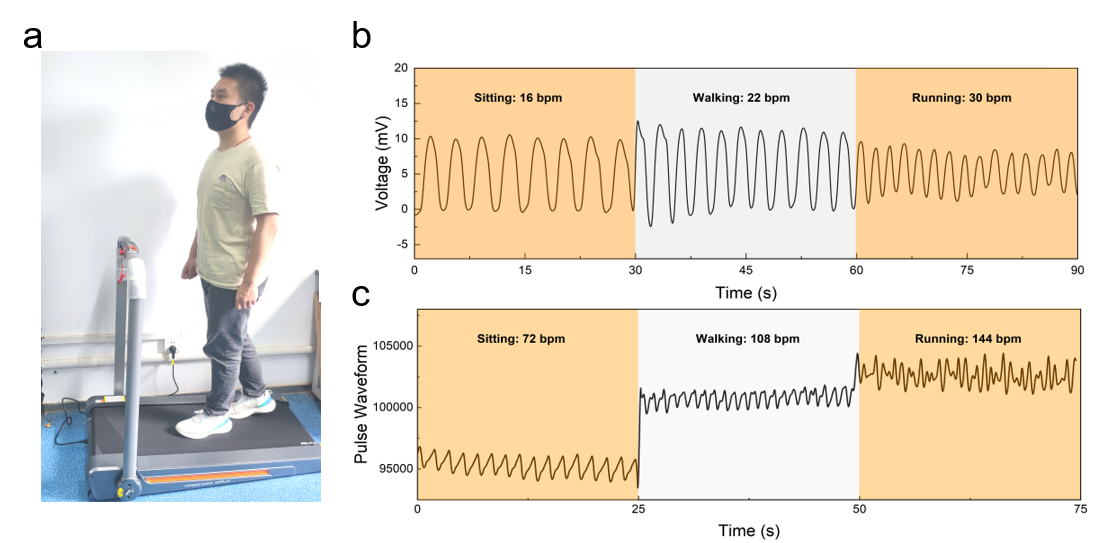


Figure S9. a) Volunteers wearing mask-like device on a treadmill. b) Respiratory waveform and respiratory rate under different states. c) Heartbeat waveform and heart rate under different states.

**Figure S9a** illustrates a volunteer wearing our wearable respiratory and heart rate monitoring system while exercising on a treadmill to test the waveform during sitting, walking, and running. Figure S9b displays the respiratory waveform under three states, showing stable patterns while sitting, increased amplitude and frequency during walking, and higher frequency with reduced amplitude during running. This is because the body requires rapid, efficient oxygen intake during running, rather than the need for the deep breaths of walking^52^. The heartbeat waveform shows increased amplitude and frequency across all three states (Figure S9c), which is due to the body's demand for more oxygen and nutrients as exercise intensity increases. The heart must pump blood more effectively, thus increasing the frequency and amplitude. This indicates that our device has excellent motion recognition capabilities, being able to stably capture signals across different states of activity.


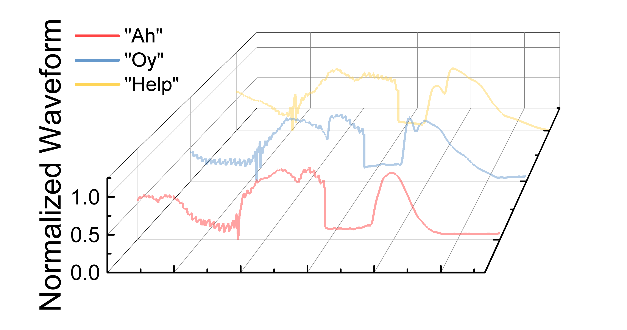


**Figure S10.** Data presentation of multi-source fusion of phrase and heartbeat.

This work utilizes the eXtreme Gradient Boost (XGBoost) algorithm to implement the recognition of abnormal vital signs. Compared to Artificial Neural Networks (ANN), the advantages of XGBoost are primarily reflected in its ability to rapidly process high-dimensional data, quick hyperparameter tuning, automatic feature selection, and lack of dependence on feature engineering. In the vast majority of tasks, XGBoost outperforms ANN. Our dataset is derived from the experiments previously collected, where heart rate and voice samples are concatenated end-to-end to form a new sample shown in Figure S10, named as multi-source fusion.

We integrated distress phrases and abnormal heartbeats through multi-source fusion, defined them as “abnormal” (negative), and the rest of the combinations were defined as “normal” (positive), and filled the data to ensure consistent data size. Cross-Entropy Loss (CEL) is usually used as the objective function of binary classification model, which is defined as:

|  | $Loss=-\frac{1}{N}\sum_{i=1}^{n} \left[ y_{i}\log\left( p_{i} \right)+(1-y_{i})log(1-p_{i}) \right]$ | (3) |
| --- | --- | --- |

Where *N* is the total number of samples, $y_{i}$ is the actual label of the *i-*th sample, and $p_{i}$ is the predicted probability of the *i-*th sample. The model hyperparameter configuration is detailed in Table 1.

Table S1. Hyperparameter configuration of XGBoost model.

| Parameter | Value | Description |
| --- | --- | --- |
| objective | binary:logistic | Specifies the objective function as binary logistic regression, used for probability prediction |
| eta | 0.01 | Learning rate, controls each tree’s contribution |
| max_depth | 5 | Maximum depth of trees, controls model complexity |
| subsample | 0.8 | Sampling ratio of training instances per tree, helps prevent overfitting |
| colsample_bytree | 0.8 | Feature sampling ratio per tree, helps prevent overfitting |
| eval_metric | logloss | Evaluation metric, using cross-entropy loss (log loss) |

The XGBoost hyperparameters used in the study are summarized in the table below. For this binary classification task (distinguishing normal from abnormal states based on voice data), the objective function was set to "binary:logistic" and the evaluation metric to "logloss." Parameters including subsample and colsample_bytree were maintained at the XGBoost default values. A relatively low max_depth was selected to enhance model generalization. The analysis employed 10-fold cross-validation, with the resulting confusion matrix showing the mean number of samples per category across folds along with standard deviations. The model achieved an overall accuracy of 99.09% ± 1.39%, indicating strong generalization performance.


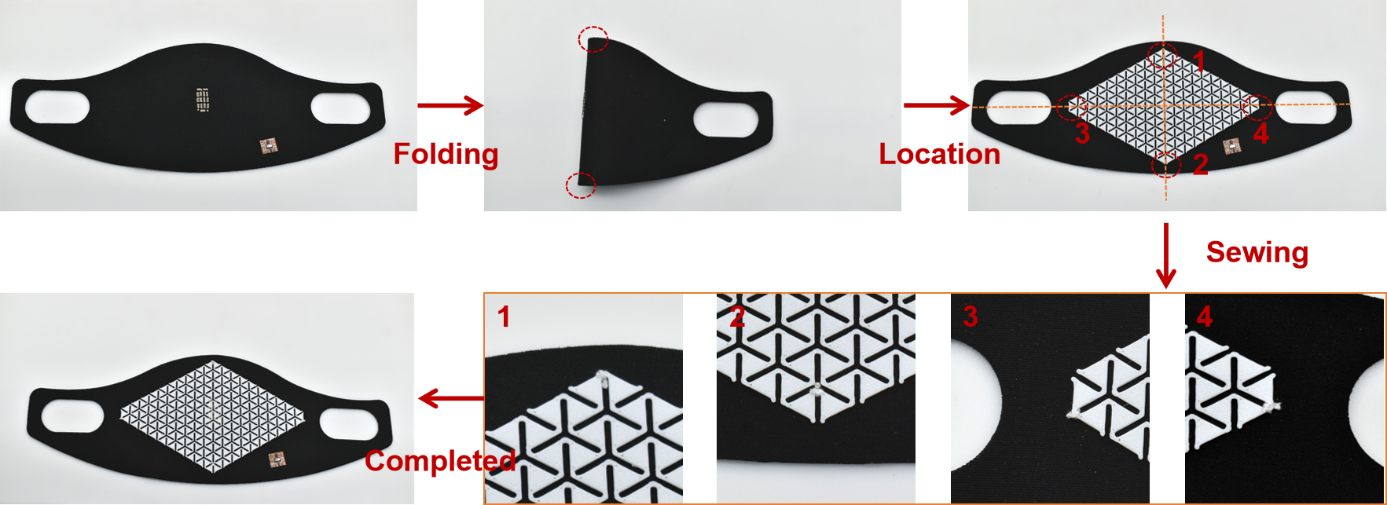


**Figure S11.** Alignment and fixation process of the Kirigami structure

First, the fabric mask undergoes a folding process. Then, location steps are carried out, utilizing marked reference points (as shown by the numbered indicators and cross - hair alignment in the "Location" figure) to ensure accurate positioning of the kirigami layer relative to the device. Finally, sewing is employed to bond the kirigami layer to the device assembly. The detailed sewing connections at different positions (shown in the close - up views of the "Sewing" step) ensure a stable and consistent bond, which is crucial for maintaining the device's performance consistency.


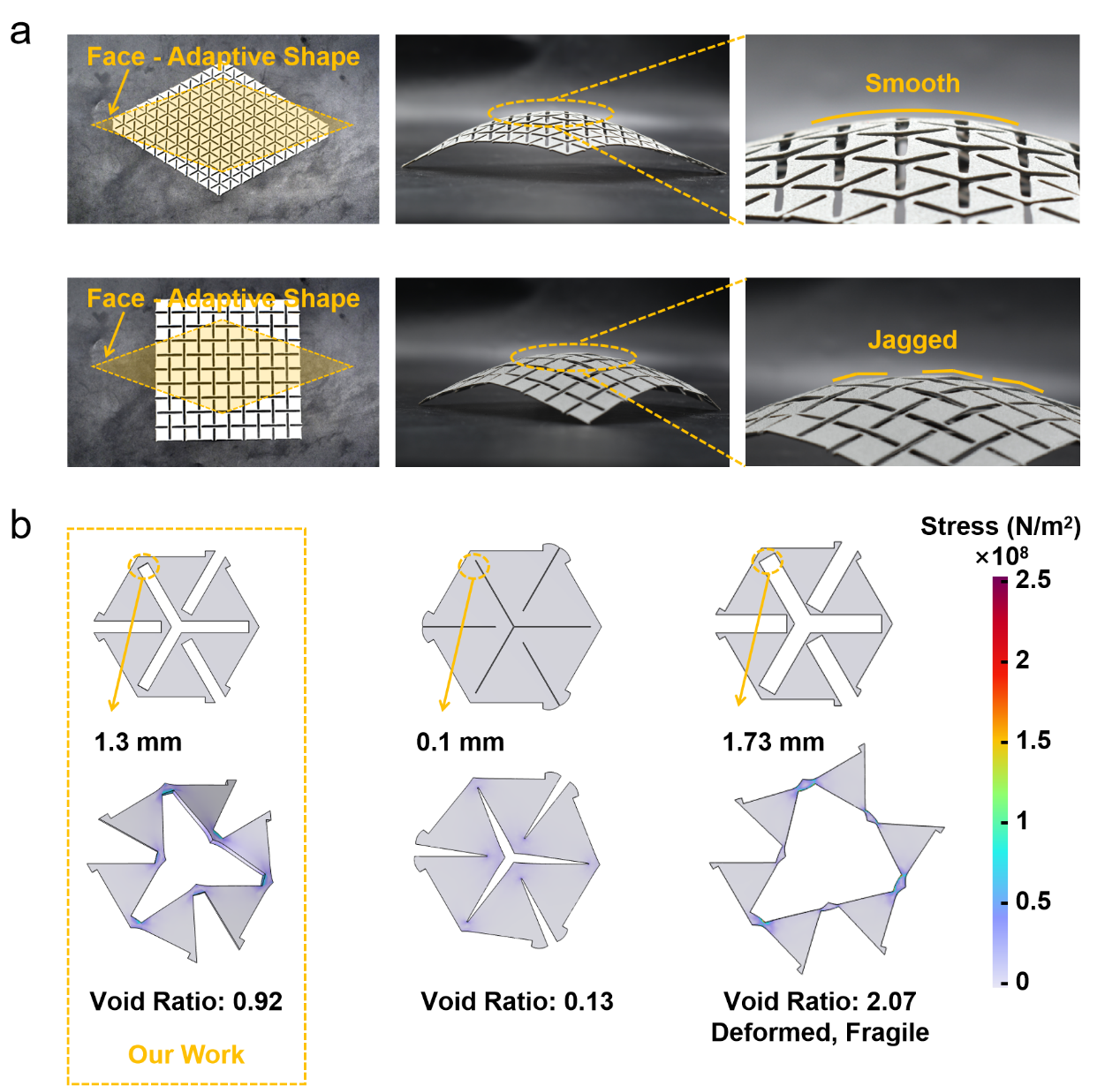


**Figure S12.** a) Comparison between triangular kirigami structures and rectangular kirigami structures. b) Simulation of spacing variation for triangular kirigami structures

To simultaneously maintain breathability and isolate the skin from the TED, we incorporated a kirigami structure as a support layer. In terms of structural selection (including cut geometry, spacing, and dimensions), we made a series of considerations. As shown in Figure a, we chose the triangular kirigami instead of the rectangular one for two reasons. Firstly, triangular kirigami can be assembled into a face - adaptive shape, while it is rather difficult for rectangular kirigami to achieve this. Secondly, the surface of stretched triangular kirigami is relatively smooth, whereas that of rectangular kirigami is rough/uneven, which is detrimental to wearing comfort.

Secondly, a series of simulations were designed to select the appropriate spacing (Figure b). We simulated triangular kirigami structures with spacings of 1.3 mm, 0.1 mm, and 1.73 mm, and applied a stress of 3.67 N. The resulting void ratios were 0.92, 0.13, and 2.07, respectively. It can be seen that the kirigami structure with a spacing of 1.3 mm has a relatively uniform stress distribution and a regular shape after stretching. Combined with the WVT test in Section 2.1, it is proved that a void ratio of 0.92 ensures good breathability. In contrast, the spacing of 0.1 mm leads to an excessively low void ratio, which is not conducive to breathability. The kirigami structure with a spacing of 1.73 mm has a void ratio of 2.07, but it is obviously severely deformed and prone to fracture. Therefore, we selected a spacing of 1.3 mm in our work. For the selection of dimensions, we adopted a moderate size (with a triangular side length of 7 mm).
